# The Shift of the Intestinal Microbiome in the Innate Immunity-Deficient Mutant *rde-1* Strain of *C. elegans* upon Orsay Virus Infection

**DOI:** 10.3389/fmicb.2017.00933

**Published:** 2017-05-29

**Authors:** Yuanyuan Guo, Zhe Xun, Stephanie R. Coffman, Feng Chen

**Affiliations:** ^1^School of Life Science, Peking UniversityBeijing, China; ^2^Central Laboratory, Peking University School of StomatologyBeijing, China; ^3^Clovis Community College, FresnoCA, United States

**Keywords:** intestinal microbiome, innate immunity, *rde-1*, Orsay virus, *Caenorhabditis elegans*

## Abstract

The status of intestinal microbiota is a determinant of host health. However, the alteration of the gut microbiota caused by the innate immune response to virus infection is unclear. *Caenorhabditis elegans* and its natural virus Orsay provide an excellent model of host–virus interactions. We evaluated the intestinal microbial community complexity of the wild-type N2 and the innate immunity-deficient mutant *rde-1* (*ne219*) strains of *C. elegans* upon Orsay virus infection. The gut microbiota diversity was decreased in *rde-1* (*ne219*) mutant animals, and a large number of genes were associated with the difference between infected and uninfected *rde-1* (*ne219*) mutant animals. Therefore, this study provides the first evaluation of the alterations caused by Orsay virus on intestinal microbiota in wildtype and innate immunity-deficient animals using *C. elegans* as the model species. Our findings indicate that virus infection may alters the microbiome in animals with defective immune response.

## Introduction

The Orsay virus was discovered in 2011 in *Caenorhabditis elegans* (*C. elegans*) nematodes in rotting fruit in Orsay, France. It is the only identified virus capable of naturally infecting *C. elegans* (Felix et al., 2011). Orsay virus has a bipartite positive-sense RNA genome and a similar genomic organization to *nodaviruses*, but differs in the presence of an additional open reading frame (ORF) in the RNA2 segment (Felix et al., 2011; Guo et al., 2014). Orsay virus-infected *C. elegans* display intestinal abnormalities, such as extensive convolutions of the apical intestinal border (Felix et al., 2011). The viral particles are mainly localized to intestinal cells (Franz et al., 2014). Remarkably, Orsay virus infection has little effect on the animal, which continues moving, eating, and producing progeny, albeit at a lower rate. Addition of dead, infected nematodes to healthy nematodes results in transmission of infection, and the presence of viral RNA in the somatic gonad suggests that Orsay virus is transmitted horizontally (Felix et al., 2011). Orsay virus is a useful model for studying host–virus interactions due to its small size, similarity to *nodaviruses*, tropism to intestinal cells of *C. elegans*, and its lack of infectivity for humans.

*Caenorhabditis elegans* is a bacterivore that ingests microbes via its pharynx, in which the process of digestion begins (Avery and Shtonda, 2003). *C. elegans* has a simple body plan with the intestine as the major body cavity open to the outside environment, so that it can directly sample the environmental microbial community (Balla and Troemel, 2013). Although *C. elegans* is a well-characterized model organism, little is known about its natural history, particularly its interactions with microbes (Petersen et al., 2015; Berg et al., 2016). Until recently, the intestinal microbiota of *C. elegans* has been characterized in the laboratory by examining worms from diverse natural soil environments. The results showed that worms harbor diverse gut microbial communities comprising 830 genus-level OTUs, 32 of which were present in all worm populations. *Enterobacteriaceae*, *Burkholderiaceae*, *Xanthomonadaceae, Pseudomonadaceae*, as well as nine other bacterial families were characterized as the worm core gut microbiota (Berg et al., 2016).

The mammalian gut microbiota influences nutrient acquisition, homeostasis stabilization, pathogen colonization, immune responses, and pathogen eradication (Endt et al., 2010; Stecher et al., 2010; Karst, 2016). Accumulating evidence suggests that the normal intestinal flora of *C. elegans* plays a critical role in maintaining a stable symbiotic relationship and is essential for several host physiological processes (Félix and Duveau, 2012; Portal-Celhay et al., 2012; Montalvokatz et al., 2013; Shapira, 2016). In a previous study, the intestinal microbiota of *C. elegans* in natural soil was identified to the species level. Two isolates, *Bacillus megaterium* (*B. megaterium*) and *Pseudomonas mendocina* (*P. mendocina*), were found to confer resistance to the nematode pathogen *Pseudomonas aeruginosa* (*P. aeruginosa*). The protection offered by *P. mendocina* was p38-dependent (Montalvokatz et al., 2013). Another recent study focused on the change in the gut microbial community after *Bacillus nematocida* (*B. nematocida*) B16 infection (Niu et al., 2016). Thus, the potential diversity of host–symbiont interactions in the gut is immense.

Furthermore, *C. elegans* is an important genetic model system for research on aging, development, and host–pathogen interactions. With the recent identification of a natural virus of *C. elegans*, this host now also represents a model for investigating virus–host interactions, particularly innate antiviral mechanisms. In the absence of a natural virus, previous studies of virus–host responses in *C. elegans* involved infection of primary cultured cells with vesicular stomatitis virus (VSV) or replication of flock house virus (FHV) in whole animals (Lu et al., 2005; Schott et al., 2005; Wilkins et al., 2005). These studies reported that the RNA interference (RNAi) pathway is involved in antiviral defense in *C. elegans* and identified genes essential for the RNAi antiviral response.

Orsay virus readily infects laboratory *C. elegans* mutants defective in RNAi and yields higher levels of viral RNA and obvious disease symptoms, as compared to infection of the corresponding wild-type N2 strain, demonstrating an antiviral role for RNAi in worms (Felix et al., 2011). Immunity to Orsay infection requires the RNAi pathway, consistent with earlier studies (Guo et al., 2012, 2013b; Ashe et al., 2013, 2015). Upon infection of *C. elegans* by Orsay virus, DRH-1 (Dicer-related RNA helicase-1) recruits Dicer-1 and the dsRNA-binding protein RDE-4 (RNA interference-deficient 4) to recognize the viral dsRNA replication intermediate. Dicer-1, with the aid of its partner RDE-4, cleaves the viral genome into 23 nt viral siRNA duplexes with a 2 nt 3′ overhang (Tabara et al., 2002; Schott et al., 2005; Wilkins et al., 2005; Ding and Voinnet, 2007; Lu et al., 2009; Ashe et al., 2013; Guo et al., 2013a). Duplex viRNAs lose one strand to produce primary viral siRNAs during incorporation into Argonaute protein RDE-1 (RNA interference-deficient 1). Subsequently, primary viRNAs are loaded into RDE-1 containing RISC complexes, which recruit the RNA-dependent RNA polymerase RRF-1 to the viral genome to synthesize secondary viral siRNA (Tabara et al., 2002; Yigit et al., 2006; Sijen et al., 2007; Steiner et al., 2009; Guo et al., 2013b). Secondary viRNAs are 22 nt single-stranded RNAs with G as the 5′-terminal, which are often referred to as 22G RNAs. These secondary siRNAs are then recruited by secondary Argonaute proteins, such as SAGO-2, and act to silence viral transcripts or inhibit virus replication (Aoki et al., 2007; Pak and Fire, 2007; Ding, 2010; Ding and Lu, 2011).

In addition to immune regulation, whether the intestinal microflora is involved in synergistic resistance to virus infection is unclear. The genes involved in the interaction with the intestinal microflora following virus infection virus remains unknown. In this study, we investigated the change in the intestinal microbiota community and the expression of related genes during the antiviral response of *C. elegans* to Orsay virus using N2 and *rde-1* (*ne219*) mutants.

## Materials and Methods

### Genetics

The Bristol isolate of *C. elegans*, N2 strain and the RNAi deficient mutant WM27 *rde-1* (*ne219*) was used in this study. All worm strains were maintained on nematode growth medium (NGM) plates at 20°C and seeded with the *Escherichia coli* (*E. coli*) bacteria strain OP50 unless otherwise indicated (Brenner, 1974). Serial passage of both *C. elegans* N2 and *rde-1* (*ne219*) strain was conducted by transferring one square centimeter of agar with mixed stage animals onto a fresh OP50 seeded NGM plate.

### Synchronization of Worm Cultures

Eggs were obtained by incubating freshly starved larvae on new 90 mm diameter seeded NGM plates with basic hypochlorite solution as previously described (Lewis and Fleming, 1995). Synchronous cultures were achieved by allowing the purified eggs to hatch overnight in M9 buffer without bacteria. The larval stage 1 (L1) animals were then washed by M9 buffer and diluted to 1000 worms per ml for Orsay virus infection.

### Infectious Filtrate Preparation and Infection Assay

Orsay virus was maintained using the JU1580 isolate of *C. elegans* at room temperature and the virus filtrate was prepared following a protocol described previously (Felix et al., 2011). To prepare Orsay virus inoculum, infected JU1580 worms were washed off from fresh slightly starved 90 mm plates using M9 buffer, 3 ml per plate. After centrifugation twice at 21000 *g* for 5 min (4°C), the virus-containing supernatant was then filtered through a 0.22-μm filter unit (Merck Millipore, Germany). For all strains, 10 young adults were inoculated on 55 mm culture plates. At the same time, 40 μl of infectious filtrate was pipetted onto the bacterial lawn. The cultures were incubated at 20°C for 4 days. Infections were performed in four biological replicates.

### DNA and RNA Extraction

Four days after infection, all worms were collected in M9 and then divided into two parallels. The worms were treated based on the method described in the literatures (Alegado and Tan, 2008; Niu et al., 2016). The worms were surface sterilized by washes with 100 mM levamisole and incubated in M9 buffer containing 100 mM levamisole and 100 mg/ml gentamicin. Levamisole treatment has been demonstrated to inhibit pharyngeal pumping and defecation. The treatment helps to prevent gentamicin entering into the intestinal lumen and the release of luminal bacteria. After incubation, the adult worms were washed with a levamisole solution to remove the gentamicin and homogenized with M9 containing 1% Triton X-100 to recover bacteria within the worm intestine.

DNA was extracted using the DNeasy^®^ Blood&Tissue Kit (QIAGEN, Germany) according to the manufacturer’s instructions. The genomic DNA was evaluated with a NanoDrop spectrophotometer (Thermo Fisher Scientific, United States), using an A260/A280 ratio between 1.8 and 2.0 as a criterion for quality control and agarose gel electrophoresis, respectively. DNA samples were stored at -20°C until use. Total RNA was prepared using Invitrogen TRIzol (Thermo Fisher Scientific, United States), by following the manufacturer’s instructions.

### Quantitative Real-Time Polymerase Chain Reaction (qRT-PCR)

cDNA was generated from 1 μg total RNA applying random primers using Superscript III (Thermo Fisher Scientific, United States), and cDNA was diluted to 1:100 for qRT-PCR analysis. qRT-PCR was performed using SYBR Green realtime PCR master Mix (TOYOBO, Japan). The amplification was performed on a CFX96 Touch^TM^ Deep Well Real-Time PCR Detection System (Bio-Rad Laboratories, Inc., United States). Each sample was normalized to *ama-1*, and then viral RNA1 (primers GW194: 59 ACC TCA CAA CTG CCA TCT ACA and GW195: 59 GAC GCT TCC AAG ATT GGT ATT GGT) levels were compared to those uninfected animals as negative controls.

### Single Molecular Fluorescent *In Situ* Hybridization Assay

Accumulation of Orsay virus and the intestinal cell infection phenotype in N2 and *rde-1* (*ne219*) mutant animals were detected by single molecular fluorescent *in situ* hybridization (smFISH), which was performed as previously described (Raj et al., 2008; Raj and Tyagi, 2010; Ji and van Oudenaarden, 2012; Franz et al., 2014). We used the set of custom Stellaris^TM^ Molecule FISH Probes (Biosearch Technologies, Inc., United States) labeled with Quasars^®^ 670 Dye for the Orsay virus RNA1 molecule in this experiment. The oligonucleotide sequences were reported by Franz et al. (2014). Color images through an Olympus BX51 DIC microscope were acquired by a Canon T1 DSLR connected to an LM Microscope adapter.

### DNA and cDNA Library Construction and Sequencing

DNA libraries were constructed according to the standard protocol provided by Illumina. Quality control of libraries were performed before sequencing. Libraries were sequenced using the Illumina Miseq instrument (Illumina, United States) with 2 × 300 base pairs (bp) paired-end (PE) sequencing.

mRNA was concentrated from total RNA and cDNA libraries were constructed using mRNA. After validation and the quality assessment of each library on gel electrophoresis and bioanalyzer (Agilent Technologies, United States), the libraries were sequenced on the Illumina Hiseq platform (Illumina, United States) with 2 × 150bp paired-end (PE) sequencing.

### DNA Library Sequencing Data Processing

The raw data generated by sequencing were analyzed using the pipeline tools in QIIME (Caporaso et al., 2010). Sequences were demultiplexed using customized Perl scripts, based on the presence of the unique barcodes assigned to each sample.

Data were de-noised by removing reads with average quality values <30. After removing both the barcodes and primer sequences, the filter-passed reads were clustered into Operational Taxonomic Units (OTUs) at a 97% similarity cutoff using the *de novo* OTU-picking strategy. Taxonomies were assigned according to the Greengenes database.

Following construction of the OTU table and phylogenetic tree, microbial richness (Chao1, observed species, and phylogenetic diversity (PD)), diversity (Shannon Index and Simpson Index), and evenness (Equitability, Simpson diversity index) estimators were calculated. Besides, microbial structure was evaluated by analyzing weighted UniFrac distance, unweighted UniFrac distance, and Bray–Curtis distance metrics (Lozupone and Knight, 2005). The relative abundances of microbial taxa at the phylum, class, order, family, genus, and species levels were calculated and compared.

### Data Processing of RNA Sequencing

Raw sequencing reads were trimmed using the following criteria: (1) reads containing adapters were removed; (2) reads containing greater than 10% N bases were removed; and (3) reads containing greater than 50% low-quality reads (*Q* ≤ 5) were removed. After which, the resulting filtered reads were mapped and annotated. Gene expression was quantified as Reads Per Kilo bases per Million reads (RPKM) (Mortazavi et al., 2008). DESeq (Anders and Huber, 2010) was used to perform statistical analyses of the gene expression profiles.

### Statistical Analysis

The data were further analyzed with the following statistical methods. (1) Differences in alpha- and beta-diversity were evaluated using the non-parametric two-sample *t*-tests with 1000 permutations. (2) Differences in relative abundances of individual OTUs and microbial taxa (phylum, class, order, family, genus, and species) were analyzed using LEfSe (Segata, 2011) with default parameters [*P* < 0.05, Linear discriminant analysis (LDA) score > 2.0].

## Results

### Orsay Virus Replicates in *C. elegans* Intestinal Cells

To monitor the composition of the intestinal microbiota of *C. elegans* upon Orsay virus infection, we analyzed 16S ribosomal DNA library data of infected and non-infected worms. The laboratory reference strain N2 and an *rde-1* (*ne219*) mutant deficient in the primary Argonaute protein RDE-1 were used in this study.

**FIGURE 1:**
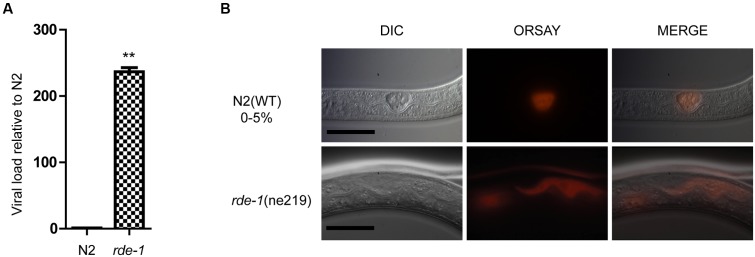
**Validation of Orsay virus replications in N2 and *rde-1* (*ne219*) mutant. (A)** qRT-PCR analysis of viral loads after 4 days Orsay virus infection in N2 and *rde-1* (*ne219*). *rde-1* (*ne219*) mutant animals showed significantly higher levels of Orsay virus RNA compared to N2 animals do (*P* < 0.01, *t*-test). **(B)** Fluorescence *in situ* hybridization (FISH) staining of Orsay viral RNA1 in *Caenorhabditis elegans* N2 and *rde-1* (*ne219*) mutant. Viral RNA1 were detected using Quasar^®^ 670 labeled RNA FISH Probe corresponding to the RNA1 of Orsay virus. Red indicates the Orsay viral RNA and all scale bars represent 20 μm.

Quantitative real-time polymerase chain reaction (qRT-PCR) and single molecular fluorescence *in situ* hybridization (smFISH) were performed to verify Orsay virus infection of *C. elegans*. *rde-1* (*ne219*) mutant animals were more sensitive to infection with Orsay virus than were N2 animals, as measured by viral load (**Figure 1A**). Previous work suggested that *C. elegans* intestinal cells are structurally altered by Orsay virus infection and the viral particles are mainly localized to intestinal cells (Felix et al., 2011; Franz et al., 2014). Using smFISH, the Orsay virus RNA1 segment was detected in intestinal cells of adult animals. Orsay virus-infected *rde-1* (*ne219*) mutant (*rde-1*_OrV) animals showed a much greater proportion of infected intestinal cells compared to N2 animals (**Figure 1B**).

### RDE-1 Deficiency Is Associated with Decreased Microbial Diversity

**FIGURE 2:**
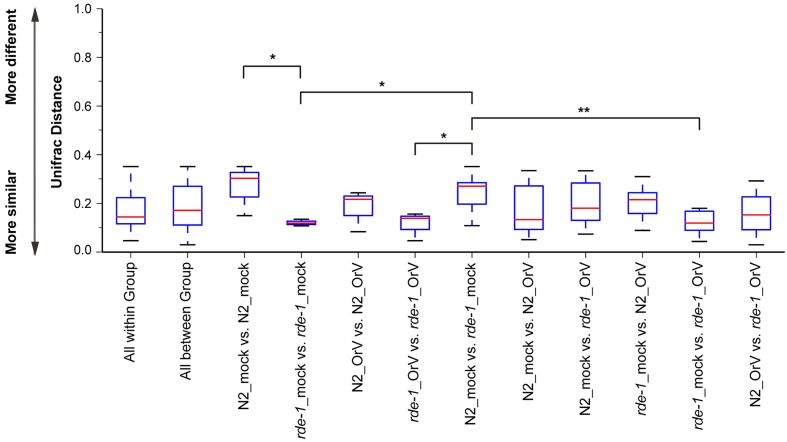
**Changes of the microbiota diversity due to Orsay infection**. The average weighted UniFrac distance values (the beta diversities) of uninfected N2 (N2_mock), Orsay virus-infected N2 (N2_OrV), uninfected *rde-1* (*ne219*) mutants (*rde-1*_mock) and Orsay virus-infected *rde-1* (*ne219*) (*rde-1*_OrV). ^∗^*P* < 0.05, ^∗∗^*P* < 0.01 by one-tailed *t*-test.

**FIGURE 3:**
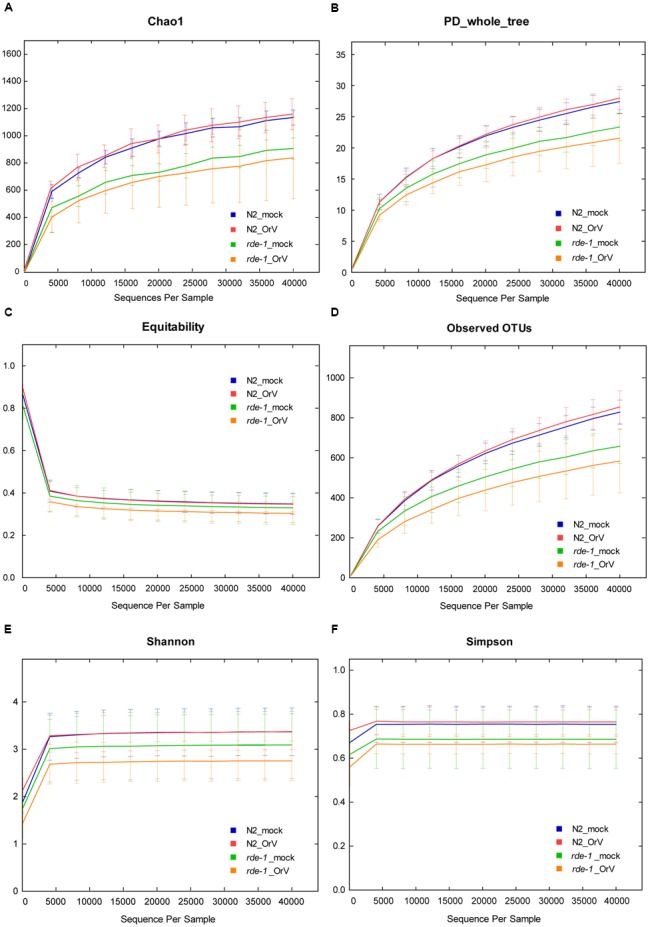
**Calculation of alpha diversity values for comparison of the total microbial diversity of uninfected and Orsay virus-infected groups of N2 and *rde-1* (*ne219*) mutant. (A)** The estimated OTU numbers (Chao1) of N2 were greater than that of *rde-1* (*ne219*) mutants, which especially decreased in Orsay virus-infected *rde-1* (*ne219*) mutants. **(B)** Phylogenetic diversity (PD) measures of community diversity. **(C)** Equitability index of the microbiota data for N2 and *rde-1 (ne219)* mutants. **(D)** The numbers of observed OTUs decreased in both uninfected and Orsay virus-infected *rde-1* (*ne219*) mutants. **(E)** Microbial community diversity analysis (Shannon index) showed that the N2 community exhibited greater diversity. **(F)** Simpson index measures of community diversity.

Orsay-infected N2 (N2_OrV) animals showed no significant difference in intestinal microbial diversity compared with uninfected animals (**Figures 2**, **3**, *P* > 0.05). According to the weighted UniFrac distance measurements, a clear separation was found between uninfected N2 (N2_mock) animals and uninfected *rde-1* (*ne219*) mutant (*rde-1*_mock) animals. The weighted UniFrac distance in N2_mock group was greater than in *rde-1*_mock group, and this demonstrated that the microbial diversity in N2 animals was significantly greater than in *rde-1* (*ne219*) mutant animals. The variation of microbial characteristics was similar for *rde-1* (*ne219*) mutant animals but significantly greater for N2 animals (**Figure 2** and Supplementary Table S1). Uninfected N2 animals showed the greatest bacterial community diversity, which was considered to be an indicator of improved health, whereas the *rde-1* mutant tended to host similar microbial communities.

We analyzed microbial diversity (alpha diversity) to determine whether RDE-1 deficiency and Orsay virus infection were associated with the intestinal microflora of *C. elegans*. The microbial diversity of N2 animals was not significantly affected by Orsay virus infection, and RDE-1 deficiency was associated with decreased microbial diversity, which decreased further after virus infection. OTU richness did not differ significantly between N2_mock and N2_OrV animals, but was lower in *rde-1* (*ne219*) mutant animals compared with N2 animals. Orsay virus-infected *rde-1* (*ne219*) mutant animals had lower OTU richness than uninfected animals (**Figures 3A,D**). The Shannon diversity indices showed that *rde-1* (*ne219*) mutant animals tended to harbor fewer diverse bacterial communities than did N2 animals, and that this decrease in diversity was exacerbated by Orsay virus infection (**Figure 3E**). The equitability index and Simpson index (measures of phylogenetic diversity, PD) indicated that the microbial communities of uninfected N2 animals exhibited the greatest diversity, and those of Orsay virus-infected *rde-1* (*ne219*) mutant animals exhibited the lowest (**Figures 3B,C,F**). Therefore, N2 animals exhibited higher levels of biodiversity and unevenness estimations compared to RDE-1-deficient animals. This indicated that RNAi plays a role in maintaining microbiomes during virus infection and the defective RNAi response in *rde-1 (ne219)* animals made the animals unable to maintain their microbiome when challenged with virus infection.

### *rde-1* (*ne219*) Mutant and N2 Animals Harbor Distinct Bacterial Communities

**FIGURE 4:**
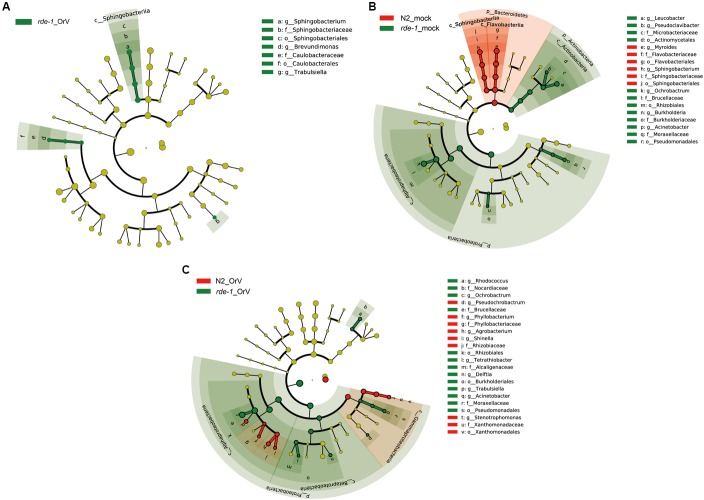
***rde-1 (ne219)* mutant and N2 animals harbor distinct bacterial communities. (A)** LEfSe analysis of microbiomes between uninfected and Orsay virus-infected group in *rde-1* (*ne219*) mutants. The taxa that were found at a significantly higher relative abundance in Orsay virus-infected group (green) relative to the uninfected samples are highlighted. **(B)** LEfSe analysis of microbiomes between uninfected N2 and *rde-1* (*ne219*) mutants. **(C)** LEfSe analysis of microbiomes between Orsay virus-infected N2 and *rde-1* (*ne219*) mutants. The taxa that were found at a significantly higher relative abundance in N2 (red) and *rde-1* (*ne219*) (green) are highlighted. For the cladogram, yellow circles represent non-significant microbial clades.

To evaluate the response of the gut microbiome to RDE-1 deficiency and virus infection, we compared the microbial clades of infected and uninfected N2 and *rde-1* mutant animals using LEfSe. The bacterial compositions differed between uninfected N2 and *rde-1* (*ne219*) mutant animals. In the latter, the gut microbiome was significantly enriched in Actinobacteria, including *Leucobacter* and *Pseudoclavibacter*; Proteobacteria, including *Acinetobacter* and *Burkholderia*; and Alphaproteobacteria, specifically, *Ochrobactrum* (**Figure 4B** and **Supplementary Figure S1A**). In contrast, the intestinal microbiome of uninfected N2 animals was enriched in Bacteroidetes, including *Myroides* and *Sphingobacterium* (**Figure 4B** and **Supplementary Figure S1A**).

To identify the microbial taxa that respond to Orsay virus infection, we examined the microbiomes of infected and uninfected *rde-1* (*ne219*) mutant animals. Orsay virus infection resulted in marked enrichment in the genera *Sphingobacterium*, *Brevundimonas*, and *Trabulsiella* (**Figure 4A** and **Supplementary Figure S1B**). However, compared with Orsay virus-infected N2 animals, the relative abundances of *Rhodococcus*, *Ochrobactrum*, *Tetrathiobacter*, *Delftia*, *Trabulsiella*, and *Acinetobacter* were higher in *rde-1*_OrV animals (**Figure 4C** and **Supplementary Figure S1C**), while the relative abundances of *Pseudochrobactrum*, *Phyllobacterium*, *Agrobacterium, Shinella*, and *Stenotrophomonas* were higher in N2_OrV animals (**Figure 4C** and **Supplementary Figure S1C**). These results suggest that Orsay virus infection and RDE-1 deficiency exert marked effects on the composition of the gut microbiome.

### Regulation of Viral Response Genes by RDE-1

To assess the relationship between the intestinal microbiome and gene expression in *C. elegans* after Orsay virus infection, we compared the mRNA profiles of mock and Orsay virus-infected N2 and *rde-1* (*ne219*) animals.

**FIGURE 5:**
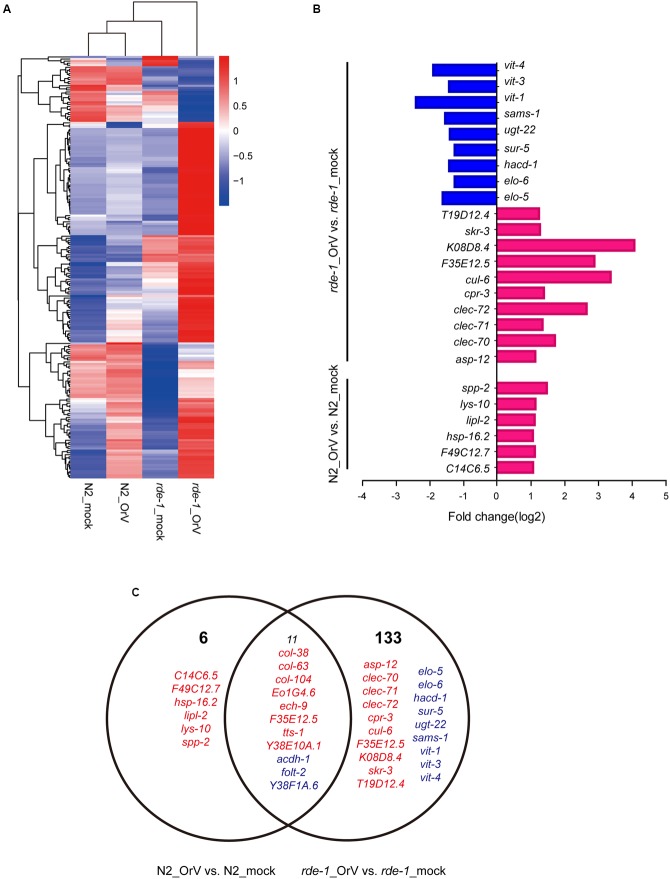
**Changes in gene expression upon viral infection. (A)** The heatmap shows all the genes that changed by more than twofold after infection (*t*-test < 0.05). Each row shows a different gene. Blue, white, and red indicate expression levels of low, medium, and high gene values, respectively. **(B)** Gene ontology (GO) and pathway enrichment analysis of predicted targets by differentially expressed mRNAs. Red indicates increased gene expression while blue indicates decreased. **(C)** The Venn diagram displaying the total number of genes significantly up-and downregulated after Orsay virus-infection in N2 and *rde-1* (*ne219*) mutant samples. Red indicates increased gene expressions, while blue indicates decreased.

The cluster analysis indicated that a large set of genes was associated with the difference between infected and uninfected *rde-1* (*ne219*) mutant animals (**Figure 5A**). We searched for genes that were significantly differently expressed (fold-change > 2 and *P* < 1 × 10^-4^, Student’s *t*-test) between uninfected and infected *rde-1* (*ne219*) animals. A total of 144 genes were specifically induced or repressed by Orsay virus infection (**Figure 5A** and Supplementary Table S2). These 144 genes showed markedly different expression levels; 126 were up-regulated and 18 down-regulated upon infection (**Figure 5C** and Supplementary Table S2). However, only 17 larger, differently expressed genes were found between uninfected and infected N2 animals; 14 genes were up-regulated and three down-regulated upon infection (**Figure 5C** and Supplementary Table S3). Gene ontology analysis showed that terms involved in innate immune response or defense response were enriched in the list of upregulated genes in infected compared with uninfected *rde-1* (*ne219*) animals (**Figure 5B** and **Supplementary Figure S2**). In contrast, genes downregulated in *rde-1* (*ne219*) animals were mainly involved with lipid metabolism, lipid transport and fatty acid metabolism (**Figure 5B** and **Supplementary Figure S3**).

## Discussion

In this study, we evaluated the complexity of intestinal microbial communities in wildtype and innate immunity-deficient mutant *C. elegans* upon Orsay virus infection. Gut microbiome diversity was decreased in *rde-1* (*ne219*) mutant animals, and several genes were associated with differences between infected and uninfected *rde-1* (*ne219*) mutant animals. Our findings indicate that the RNAi pathway may play a role in maintaining the microbiome during virus infection.

Orsay virus infection of N2 animals did not significantly influence microbial diversity, while *rde-1* deficiency was associated with decreased microbial diversity, which was further reduced by Orsay virus infection. The gut microbiota status is a determining factor of host health. The intestinal microbial diversity of the *rde-1* (*ne219*) mutant was further reduced, suggesting that the mutant animals were unstable and easily disturbed by external factors. Our results are consistent with a previous report of microbial enrichment and complexity in healthy hosts (Niu et al., 2016).

The complex intestinal microbial community is regulated by environmental, diet and host genetic factors (Kau et al., 2011; Sommer and Bäckhed, 2013). The composition, density, and complexity of the intestinal microbiota influence pathogen colonization, immune responses, and pathogen clearance (Endt et al., 2010; Stecher et al., 2010; Portal-Celhay et al., 2012; Cabreiro and Gems, 2013). In this study, we compared the intestinal microbiome in the presence and absence of Orsay virus using LEfSe. Compared to the control group, infected animals had a significantly greater number of OTUs related to the genera *Sphingobacterium*, *Brevundimonas*, and *Trabulsiella*. However, compared with uninfected *rde-1* (*ne219*) mutant animals, *Sphingobacterium* was enriched in uninfected N2 animals, suggesting that Orsay virus infection and genetic factors influence the microbial community. *Sphingobacterium* is characterized by high concentrations of sphingophospholipid components (Yabuuchi et al., 1983). *Sphingobacterium* is abundant in cowpea root nodules and significantly influenced by soil type and plant genotype (Leite et al., 2016). *Trabulsiella* strains have been found in the termite gut and may play roles in carbohydrate metabolism and aflatoxin degradation. Furthermore, *Trabulsiella* strains are adapted to their termite host, such as by expressing genes encoding the components of a type VI secretion system (T6SS), which facilitates bacterial competition, colonization, and survival within the host (Chou et al., 2007; Sapountzis et al., 2015; Suman et al., 2016).

To investigate the changes in the intestinal microbiota community and the expression of related genes in *C. elegans*, we analyzed mRNA profiles of mock and Orsay virus-infected N2 and *rde-1* (*ne219*) mutant animals. Several genes were specifically induced or repressed by Orsay virus infection. *acdh-1*, a “dietary sensor” (Kawasaki et al., 2013; MacNeil et al., 2013), was significantly down-regulated in Orsay virus-infected N2 animals, and further decreased in *rde-1* (*ne219*) mutant animals. This indicated that stress response genes were stimulated by virus infection and possibly associated with the immune response. In the presence of Orsay virus, genes related to the innate immune response, such as a cluster of C-type lectin (CLEC), were significantly up-regulated in the *rde-1* (*ne219*) mutant animals. CLEC, which contribute to immune specificity in both vertebrates and invertebrates, maintain gut homeostasis by symbiotic microbiome offsets of the gut immunity in mosquitoes (Schulenburg et al., 2008; Pang et al., 2016). The gut microbiome of mosquitoes could induce the expression of CLEC, which coat the bacterial surface to counteract the activity of antimicrobial peptides (AMPs) (Pang et al., 2016). The genome-wide associations of CLEC gene clusters suggest that CLEC genes are associated with human gut microbiome composition and function (Bonder et al., 2016). The up-regulation of *clec-70*, *clec-71*, and *clec-72* in infected *rde-1* (*ne219*) mutant animals indicated that the CLEC genes may contribute to intestinal microbiome alternation associate with virus infection in *C. elegans* with defective immune response. Moreover, multiple genes were down-regulated in *rde-1* (*ne219*) mutant animals, which highlights the role of sensor molecules in lipid metabolism, lipid transport and fatty acid metabolism, particularly vitellogenins (VITs). Lipid metabolism is an important pathway associated with aging (Nakamura et al., 1999; Gubert et al., 2016). Besides providing energy for developing embryos, VITs are involved in the stress response in diverse organisms (Fischer et al., 2012). VITs were found to act as acute- phase-proteins and to possess bacterial binding and inhibiting activities in different fish-species (Zhang, 2011; Fischer et al., 2013). VITs increase the stress resistance of *C. elegans* after *Photorhabdus luminescens* infection in a manner dependent on the steroid-signaling pathway (Fischer et al., 2013). The down-regulationof *vit-1*, *vit-2*, and *vit-4* in Orsay virus-infected *rde-1* (*ne219*) mutant animals indicates that VITs may be involved in the interactions among host genetic factors, virus infection and the microbiome.

The intestinal microbiota plays a stimulatory role in viral infection (Karst, 2016). However, the effect on the gut microbiota of virus infection remains unclear, particularly in *C. elegans.* This study reports for the first time the effect of Orsay virus infection on the intestinal microbiome and expression of related genes of N2 and *rde-1* (*ne219*) mutant *C. elegans*. Our results demonstrate that intestinal microbiota diversity may be associated with host genes during virus infection. Consistent with a previous report that host genotype affects the intestinal microbiota, our data may help to explain human diseases caused by different genotypes associated with a variety of intestinal microbiome. Furthermore, our results may lead to a better understanding of the mechanism and evolution of the mutualistic relationship between a host and its gut microbial community based on nematode molecular genetics and immunity.

## Author Contributions

YG contributed to design all the experiments, bench work and draft the manuscript. ZX contributed to analyze data. SC contributed to smFISH experiment. FC contributed to design all the experiments and draft the manuscript.

## Conflict of Interest Statement

The authors declare that the research was conducted in the absence of any commercial or financial relationships that could be construed as a potential conflict of interest.

## Supplementary Material

FIGURE S1 **Differentially abundant microbial clades in N2 and *rde-1* mutant animals upon Orsay virus infection**. (A) Linear discriminant analysis (LDA) score of microbiomes between uninfected N2 and *rde-1* mutant. (B) LDA score of microbiomes between Orsay virus-infected N2 and *rde-1* mutant. Taxa that are found at a significantly higher relative abundance in N2 (red) and *rde-1* (green) are highlighted. (C) LDA score of microbiomes between uninfected and Orsay virus-infected group in *rde-1* mutant. Taxa that are found at a significantly higher relative abundance in Orsay viurs-infected group (green) relative to the uninfected samples are highlighted.

FIGURE S2 **Gene ontology (GO) and pathway enrichment analysis of differentially upregulated expression mRNAs in *rde-1* mutant upon Orsay virus infection**. The vertical axis shows the enriched GO term, and the horizontal axis represents the number of differentially expressed genes in the term. With “*” for significantly enriched GO term.

FIGURE S3 **Gene ontology (GO) and pathway enrichment analysis of differentially downregulated expression mRNAs in *rde-1* mutant upon Orsay virus infection**. The vertical axis shows the enriched GO term, and the horizontal axis represents the number of differentially expressed genes in the term. With “*” for significantly enriched GO term.
