# Supplementary figures and images for: The Shift of the Intestinal Microbiome in the Innate Immunity-Deficient Mutant rde-1 Strain of C. elegans upon Orsay Virus Infection
Source: Front Microbiol. 2017 May 29;8:933. doi: 10.3389/fmicb.2017.00933 (PMC5446984; doi:10.3389/fmicb.2017.00933)

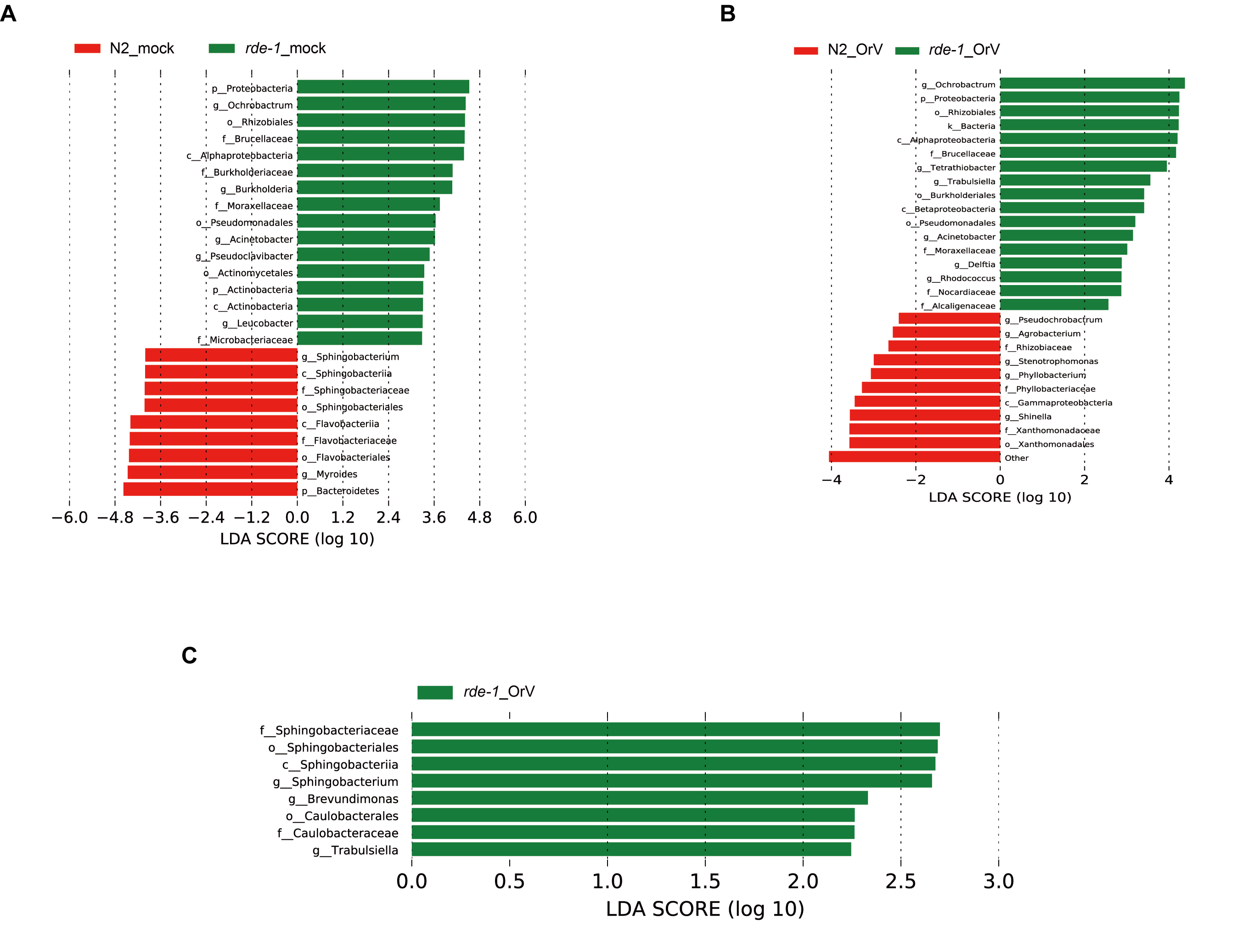

Supplement: FIGURE S1 — Differentially abundant microbial clades in N2 and rde-1 mutant animals upon Orsay virus infection. (A) Linear discriminant analysis (LDA) score of microbiomes between uninfected N2 and rde-1 mutant. (B) LDA score of microbiomes between Orsay virus-infected N2 and rde-1 mutant. Taxa that are found at a significantly higher relative abundance in N2 (red) and rde-1 (green) are highlighted. (C) LDA score of microbiomes between uninfected and Orsay virus-infected group in rde-1 mutant. Taxa that are found at a significantly higher relative abundance in Orsay viurs-infected group (green) relative to the uninfected samples are highlighted. [file Image_1.TIF]

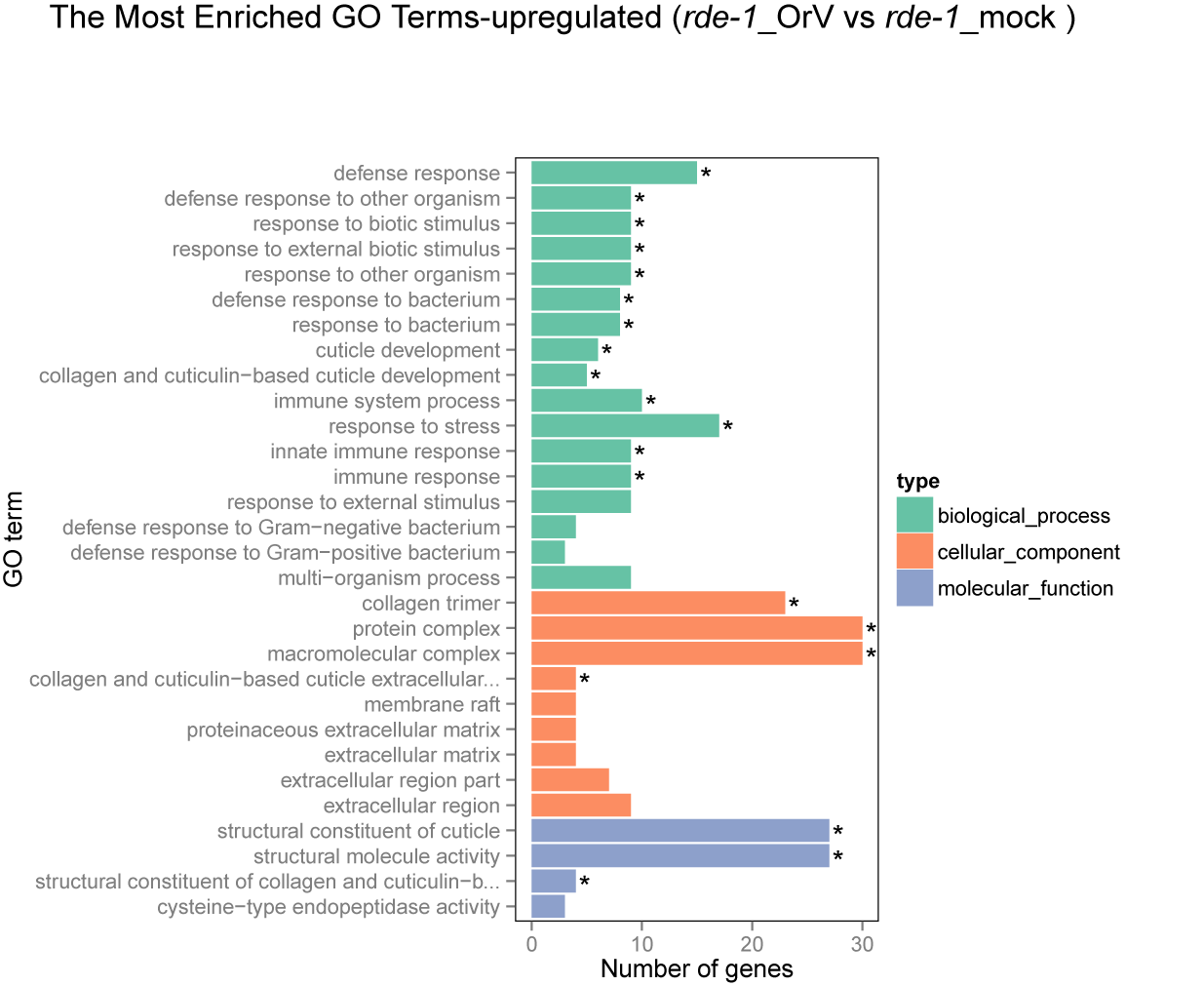

Supplement: FIGURE S2 — Gene ontology (GO) and pathway enrichment analysis of differentially upregulated expression mRNAs in rde-1 mutant upon Orsay virus infection. The vertical axis shows the enriched GO term, and the horizontal axis represents the number of differentially expressed genes in the term. With “*” for significantly enriched GO term. [file Image_2.TIF]

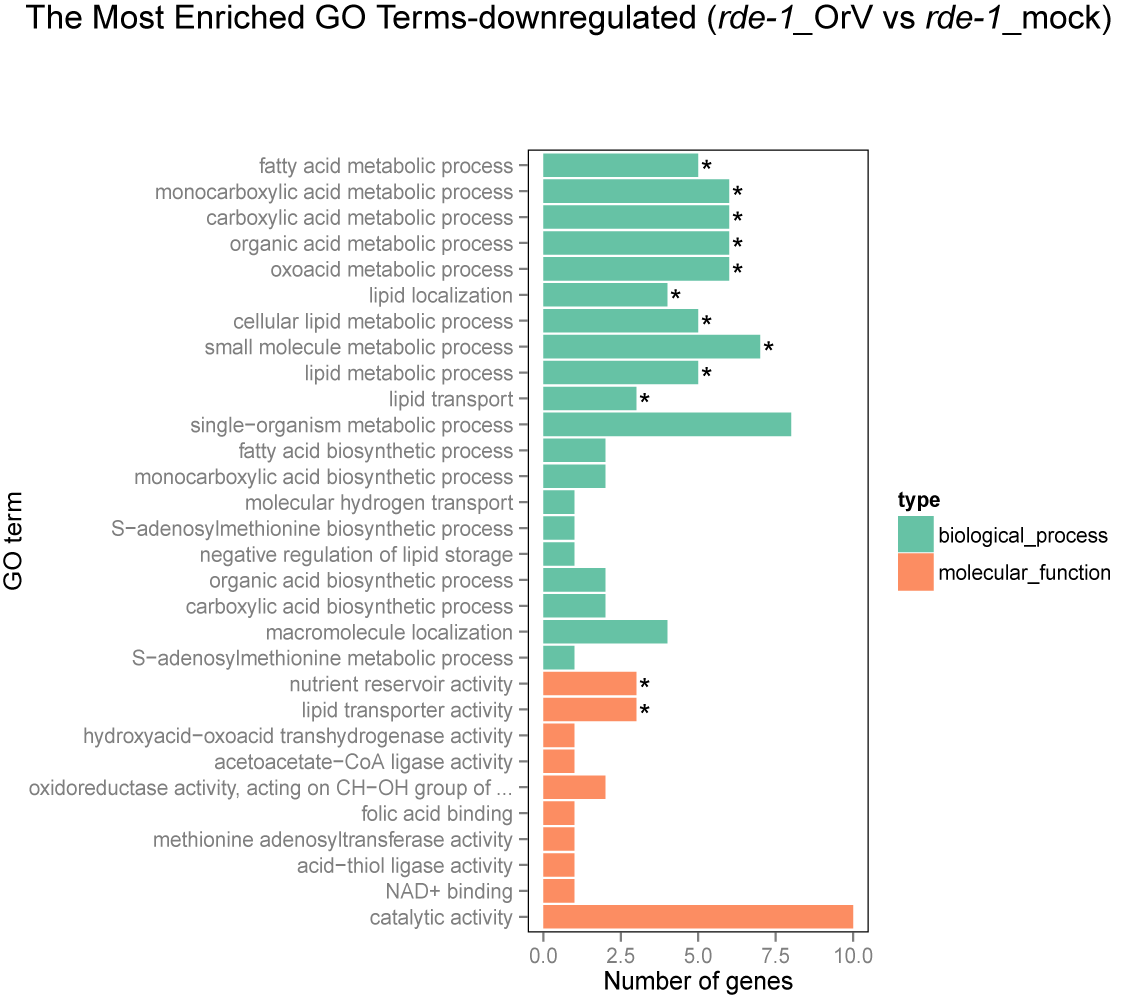

Supplement: FIGURE S3 — Gene ontology (GO) and pathway enrichment analysis of differentially downregulated expression mRNAs in rde-1 mutant upon Orsay virus infection. The vertical axis shows the enriched GO term, and the horizontal axis represents the number of differentially expressed genes in the term. With “*” for significantly enriched GO term. [file Image_3.TIF]
