# Supplementary material for: The Shift of the Intestinal Microbiome in the Innate Immunity-Deficient Mutant rde-1 Strain of C. elegans upon Orsay Virus Infection
Source: Front Microbiol. 2017 May 29;8:933. doi: 10.3389/fmicb.2017.00933 (PMC5446984; doi:10.3389/fmicb.2017.00933)
Supplement: Supplementary file 7 [file Data_Sheet_1.DOCX]

**Supplementary figure legends**

**Supplementary Fig. S1. Differentially abundant microbial clades in N2 and rde-1 mutant animals upon Orsay virus infection.** (A) LDA score of microbiomes between uninfected N2 and *rde-1* mutant. (B) LDA score of microbiomes between Orsay virus infected N2 and *rde-1* mutant. Taxa that are found at a significantly higher relative abundance in N2 (red) and *rde-1* (green) are highlighted. (C) LDA score of microbiomes between uninfected and Orsay virus infected group in *rde-1* mutant. Taxa that are found at a significantly higher relative abundance in Orsay infected group (green) relative to the uninfected samples are highlighted.

**Supplementary Fig. S2. Gene ontology (GO) and pathway enrichment analysis of differentially upregulated expression mRNAs in *rde-1* mutant upon Orsay virus infection.**

The vertical axis shows the enriched GO term, and the horizontal axis represents the number of differentially expressed genes in the term. With "*" for significantly enriched GO term.

**Supplementary Fig. S3. Gene ontology (GO) and pathway enrichment analysis of differentially downregulated expression mRNAs in *rde-1* mutant upon Orsay virus infection.**

The vertical axis shows the enriched GO term, and the horizontal axis represents the number of differentially expressed genes in the term. With "*" for significantly enriched GO term.
